# Post-Marketing Experience of Edaravone in Amyotrophic Lateral Sclerosis: A Clinical Perspective and Comparison With the Clinical Trials of the Drug

**DOI:** 10.7759/cureus.10818

**Published:** 2020-10-06

**Authors:** Juan Fernando Ortiz, Sawleha Arshi Khan, Amr Salem, Zayar Lin, Zafar Iqbal, Nusrat Jahan

**Affiliations:** 1 Neurology, California Institute of Behavioral Neurosciences & Psychology, Fairfield, USA; 2 Research, California Institute of Behavioral Neurosciences & Psychology, Fairfield, USA; 3 Hospital Medicine, California Institute of Behavioral Neurosciences & Psychology, Fairfield, USA; 4 Internal Medicine, California Institute of Behavioral Neurosciences & Psychology, Fairfield, USA; 5 Emergency Medicine, California Institute of Behavioral Neurosciences & Psychology, Fairfield, USA; 6 Emergency Department, The Kidney Center, Karachi, PAK

**Keywords:** amyotrophic lateral sclerosis, edaravone.

## Abstract

Amyotrophic lateral sclerosis (ALS) is a rapidly progressive neurodegenerative disease that affects the upper and lower motor neurons. Currently, the only treatment for ALS is riluzole, which only has a limited effect on increasing survival from 3 to 6 months. New therapies are needed in the clinical setting for ALS. We aim to compare and contrast the clinical trials of edaravone and the post-marketing experience of the drug during this study. For the method, a search strategy was made using PubMed with the search terms "Amyotrophic lateral sclerosis" (MeSH) and "Edaravone" (MeSH). For inclusion criteria, we used full papers, studies involving humans, and studies published in the English language. We exclude meta-analyses, literature reviews, systematic reviews, studies involving animals, and studies not published in English. After close examination, 20 papers were used for the discussion in this review. The clinical trials showed efficacy in patients in reducing the revised ALS functional rating scale (ALSFRS-R) in patients with early ALS with selective criteria. We documented edaravone's post-marketing experience in six countries: Kuwait, South Korea, Argentina, United States, Israel, and Italy. During the study we analyzed, the forced vital capacity (FVC) and ALSFRS-R scored, together with edaravone's safety in the clinical trials and post-marketing experience. Edaravone seems to be more effective in Asia, where the ALSFRS-R scores and the FVC decline were similar to the clinical trial results in Japan. Studies in Europe did not find the drug clinically useful. At the same time, studies in United States and Argentina were mainly descriptive, so more information is needed to evaluate the drug's efficacy in that part of the world. The drug was well-tolerated in all studies. In conclusion, more studies need to be done worldwide to carry out and clarify the effectiveness of edaravone in the clinical setting.

## Introduction and background

Amyotrophic lateral sclerosis (ALS) is a neurodegenerative disease affecting upper motor neurons and lower motor neurons, leading to severe disabilities and rapid death post-diagnosis [1]. The incidence and prevalence of ALS are one to 2.6 cases per 100.000 persons annually and six cases per 100.000 persons, respectively. Metals, military service, and smoking have been shown to increase ALS risk [1]. Although the pathogenesis of ALS continues to be mainly unknown, the central hypothesis is the inflammation, excitotoxicity, and defects in the intracellular and nucleocytoplasmic transport as well as oxidative stress [2]. Currently, the primary drug used for the treatment of ALS is riluzole. This drug inhibits glutamate release from presynaptic terminals and blocks the post-synaptic N-methyl-D-aspartate (NMDA) receptors, which have been shown to increase survival by three to six months on average [3]. At the moment, ALS has more symptomatic treatment with a multidisciplinary approach. For this reason, new therapies are needed to treat ALS.

In 2017 the FDA approved edaravone for the treatment of ALS. Edaravone appears to play a role in oxidative stress, which has been shown to be a part of the onset and progression of the disease [4]. Edaravone is a free radical scavenger that lowers the neuronal damage, eliminating the lipid peroxide hydroxyl radicals and transfers the electrons to edaravone (the radical) to ameliorate the oxidative damage [5]. Since 2006, many clinical trials of ALS have been conducted. Initially, studies showed decreased markers of oxidative stress in the cerebrospinal fluid (CSF) and a positive tendency of decreasing the scores of the revised amyotrophic lateral sclerosis functional rating scale (ALSFRS-R) [6]. The initial phase III study failed to prove statistical significance between the placebo group and the edaravone group [7]. Nevertheless, post-hoc analyses showed that patients with early symptoms of ALS were more prone to respond to edaravone [8]. These studies have faced criticism because they did not demonstrate that the drug increases survival, in part because a functional scale was used instead of a survival scale. Another problem is that the studies were developed in Japan, so studies around the world might draw different conclusions with a more diverse population [5]; the clinical trials have not studied the interaction of riluzole and edaravone for the treatment of ALS. Edaravone could potentially have an additive effect on riluzole. Studies have demonstrated efficacy only in a small group of patients with early ALS, so new information in a bigger and more diverse pool of patients might draw new conclusions [8].

Edaravone is a new drug on the market for ALS treatment, so we aim to consolidate the most important clinical findings during the trials of edaravone and compare those findings with the post-marketing experience. It is imperative to analyze the efficacy and safety of the drug as well, after the clinical trials. Because the genetics in ALS may vary in different populations [9], it is essential to review how the post-marketing experience in other parts of the world may vary and to determine how its efficacy and safety may change depending on the region. The parameters we plan to analyze and discuss during this review are ALSFR-R score, forced vital capacity (FVC), survival rate, and the possible additive effect of edaravone on riluzole.

## Review

Method

Data was collected on PubMed using the following medical subject heading (MeSH) terms: "amyotrophic lateral sclerosis" (MeSH) and "edaravone" (MeSH). Table 1 exhibits the regular and MeSH keywords utilized for the review. 

**Table 1:** Regular and MeSH keywords for literature search. MeSH: Medical subject headings.

"Amyotrophic Lateral Sclerosis" (MeSH) and "Edaravone" (MeSH)	
Total records	51
Records selected	20

Inclusion Criteria

Studies were selected after applying the following inclusion criteria: 

(1) Full papers.

(2) Studies published in English.

(3) Studies conducted on humans.

(4) All types of investigations including clinical trials, observational studies, comments, editorials, and journal articles.

Exclusion Criteria

Studies were selected with the following exclusion criteria: 

(1) Studies involving animals.

(2) Papers published in languages other than English.

(3) Study types that were case reports, literature reviews, and systematic reviews.

Results

 Table 2 exhibits the number of papers after applying the inclusion/exclusion criteria in the sequential order of the criteria used. 

**Table 2:** The inclusion/exclusion criteria used for the paper in the sequential order. MeSH: Medical subject headings.

Inclusion/Exclusion Criteria	
Total records	51
Full text papers	50
Studies on humans	49
English language	47
All types of investigations including clinical trials, observational studies, comments, editorials, and journal articles.	41
Exclude study types that were case reports, literature reviews, and systematic reviews.	33

**Figure 1:**
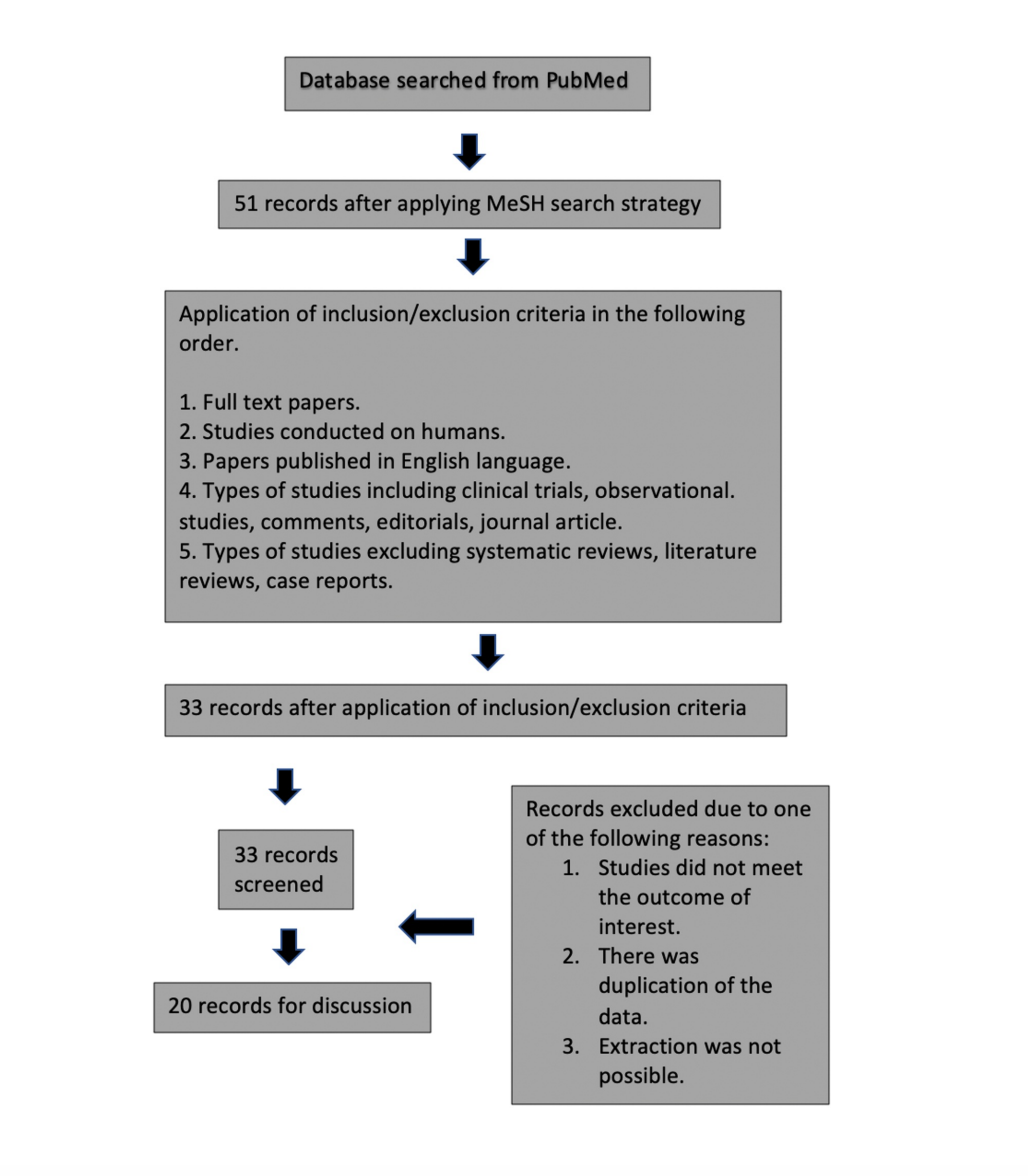
Flowchart with the method applied and the results obtained during the review.

A total of 33 papers were selected after applying the inclusion/exclusion criteria. After this first selection of papers, an initial screening was conducted based on the eligibility of titles and abstracts. The 33 articles were reviewed carefully by each author of this paper. After a refined search, we selected 20 papers for discussion by excluding 13 papers due to one of the following reasons (Figure 1): 

1. Studies did not show the desired outcome. 2. Data extraction was not possible. 3. There was duplication of data. 

For the discussion, we use the following types of studies: 

· Eleven clinical trials (discussion of the clinical trials) · Seven open-label studies (discussion of the post-marketing experience) · One editorial · One comment

Discussion 

Review of the Clinical Trials

A phase II clinical trial was conducted to determine the efficacy of a 60-mg dose of edaravone. The primary endpoint was the change in the revised ALSFRS-R score, while the secondary endpoint was 3-nitrotyrosine (3NT) level in CSF. After six months, there was a reduction in the ALSFRS-R scores and 3NT levels. Overall, the drug was well-tolerated [10]. Another phase II clinical trial with 26 patients had a significant decrease in the free acid levels and other oxidative markers when using edaravone compared to placebo, showing its antioxidant effect [11]. After these trials, a phase III clinical trial, MCI-186 study, was conducted with 306 patients with ALS to confirm the efficacy and safety of the phase II clinical trial of edaravone. The study did not show statistical significance between the placebo and the edaravone group in the decline rates of ALSFR-R; however, a good tendency was noted in the MCI-186 study [7].

The patients of the MCI-186 study did not have very selective criteria (age 20-75 years, FVC of at least 70%, disease duration of three or more years with ALS, and have the diagnosis according to revised Airlie House diagnostic criteria). New post-hoc studies were conducted because of the MCI-186 study's lack of success with patients in early-stage ALS with the following criteria: 

a. Patients having FVC values of more than 80%. b. Disease duration of two years or less, and scores ALS of grade one or two in the Japan ALS severity classification. c. Scores of at least two points on all 12 items of ALSFRS-R. d. Patients having fewer than at least two points on all 12 items of ALSFRS-R [8,12].

In the first post-hoc study, the primary outcome showed a significant reduction in the ALSFRS-R score over the placebo group. There were no significant differences in side effects between the placebo and drug treatment group [12]. The second post-hoc analysis, MCI-186-19 study, showed that edaravone favored each of the ALSFR-R score domains. There were similar effects of the drug between motor and bulbar onset ALS patients. This study showed that edaravone was beneficial in patients with early-stage ALS [8].

The MCI-186 study had an extension study, MCI-186-17. During this study, three subpopulations were defined: (1) Full analysis set (FAS) group, defined by the patients with the criteria of study MCI-186; (2) efficacy-expected sub-population (EESP), defined by scores of two points in all 12 items of the ALSFRS-R, FVC more than 80% at baseline; and (3) definite/probable EESP for two years (dpEESP2y), defined with the same criteria of EESP plus having definite or probable ALS diagnosed by El Escorial-revised criteria and having a disease duration of two years or more [13]. In study MCI-186-17, the variation in the ALSFRS-R score and FVC between 24 and 48 weeks was measured. Patients who met the dpEESP2y criteria were used for the analysis. The three groups of patients were analyzed in this study. Patients who received the drug in the previous study were reassigned to receive either 60 mg of edaravone again (E-E group) or switched to placebo (E-P group), and patients who were on placebo were switched to edaravone (P-E group). During the study, the ALSFRS-R and FVC were analyzed. All three groups showed beneficial improvement over 24-48 weeks; all groups favored edaravone over placebo [13]. The difference in ALSFRS-R scores from 24 to 48 weeks between the E-E group and E-P groups in the dpEESP2y was 2.79 (p = 0.0719), which was more significant than the previous differences between the EESP and the FAS group [13]. 

A parallel study of the MCI-186 study with a different group of patients was conducted. Patients had less selective criteria as compared with the other mentioned post-hoc analysis. In the study, patients in the early and late stages of ALS were included. In the end, there was no statistically significant difference in the changes of ALSFR-R score over the placebo group, confirming the lack of efficacy of edaravone in the later stages of the disease [14]. In a different parallel study, an extension of the first phase III clinical trial MCI-186 was made. Patients who were taking edaravone for 24 weeks were given the same drug for an additional 24 weeks, E-E group, or switched to placebo, E-P group, while patients receiving placebo in the first phase of the study were switched to the edaravone P-E group. ALSFRS-R scores between the FAS group and EESP groups were measured. At the end of the study, the decline in the ALSFRS-R scores was lower in the E-E group than the P-E group or the E-P group [15]. An extension of study MCI186-19 was conducted to prove sustained efficacy past 24 weeks. The initial study lasted 24 weeks, and there was an open-label extension of 24 weeks to see if there was a sustained benefit from the initial 24-week period. Patients who received edaravone continued receiving edaravone, E-E group, and those receiving placebo were switched to edaravone, P-E group. Patients who received edaravone for 48 weeks in the E-E group had a less functional decline in the ALSFR-S scores over the patients who received 24 weeks of edaravone after being on placebo and P-E group. They concluded that edaravone in the early phase has an extended effect past 24 weeks [16].

 In another post-hoc analysis, the ALSFRS-R score was projected, and it was found that the projected ALSFRS-R score was significantly lower for the edaravone treatment group than the placebo group, which confirmed the added benefit of edaravone over 24 weeks [17]. Finally, in another study, two subgroups of patients, EESP and dpEESP2y, were analyzed from MCI-186. An analysis of variance (ANOVA) analysis showed a significant difference in the ALSFR-R scores between the edaravone groups EESP and dpEESP2y compared to the FAS group, which was the population of patients of the MCI-186 study. The difference is even more significant in the dpEESP2y than the EESP compared to FAS. Differences in FVC% and modified Norris scale scores were also significant. Differences in FVC% were significant as well when compared to the FAS group. The dpEESP2y group showed higher efficacy in all the parameters for the drug. There were no significant differences between the side effects of the EESP and dpEESP2y groups and placebo [18].

Post-Marketing Experience 

United States

A survey of 67 physicians in the United States reported 3007 patients that were prescribed with edaravone, where 67% of patients were using edaravone in conjunction with riluzole, 24% of patients used riluzole at some point, and 9% were using riluzole naive. Fifty-nine percentage of the patients who received the drug had an implanted port, 21% utilized a peripherally inserted central catheter, and 18% used a peripheral line. Forty-three percentage of patients received edaravone at home, while others received it at clinics. Drug ineffectiveness was reported in more than 50 cases. Overall, edaravone was well-tolerated except for minor side effects such as asthenia, fatigue, gait disturbance, disease progression, muscular weakness, and dyspnea in a few cases. Only one patient reported anaphylaxis with an infusion [19].

Argentina

The use of edaravone is limited in Argentina, mainly because the insurance companies do not cover it. The study describes its use in 16 patients (14 limb onset and two bulbar onsets). Patients were treated from 2016 to April 2020. During the research, they analyzed the characteristics of the patients treated with edaravone; there were no criteria for selecting patients. All patients except one were receiving riluzole at the same time. The mean time of diagnosis of ALS before receiving treatment with edaravone infusion was 19.8 months. One patient suffered thrombophlebitis, and another suffered limb pain with infusion. Besides those events, most of the patients had good tolerance for the drug. Two patients stopped the medication due to dissatisfaction. Four patients underwent a tracheostomy, and one of the patients died [20].

Korea 

Following the El Escorial criteria, FVC and ALSFR-R scores showed modest results as compared with the clinical trials in Japan [3]. The phase III clinical trial showed a mean decrease of −14.57 ± 2.41 in FVC% after six months and a mean reduction of −5.70 ± 0.85 in the ALSFR-R scores after six months [7]. In the Korean study, the reduction in six months was lower, with 8.7%. The ALSFR-R scores in the limb onset patients and bulbar onset patients were 3.92 ± 10.01 and 11.25 ± 2.86, respectively, in the ALSFRS-R scores. Overall, there was a modest effect of edaravone on the Korean population. Minor side effects were reported in three patients, and 16% of patients reported severe adverse events [21]. No deaths were found during the Korean study. During the Korean study, the inclusion and exclusion criteria where edaravone was effective were not used [21].

Kuwait

The study included 17 patients with ALS (12 limb onset and five bulbar onset) who fulfilled the criteria of the MCI-186-J19 study [8]. This study was a prospective observational cohort study treated with edaravone's infusion in Kuwait and was assessed at 24, 28, and 72 weeks. The study's primary outcome was the rate of decline of ALSFRS-R and FVC. Other assessed points were the need for tube feeding, non-invasive ventilation, tracheostomy tube, mechanical ventilation, ambulation, survival rate, and satisfaction with the drug. Riluzole intake or other ALS treatments were allowed in the study, and there was no control group for comparison. This study's participants had the same criteria as study MCI-186-J19, where edaravone proved to be effective [8]. A study decline was observed in the parameters of the primary outcome. The decrease in ALSFRS-R parameters was 1.6 ± 1.9 after 24 weeks versus 5.7 and 5.01 in the MCI-186 and MCI-186-J19 studies, respectively [7,8]. The decline at 48 weeks was 3.8 ± 3 versus 8 points in the MCI-186-J19 study [8,22]. The reduction at 72 weeks was 7.9 ± 6.6, suggesting decreased efficacy after 48 weeks of use. It is important to note that the clinical trial did not evaluate the drug's effectiveness after 48 weeks. FVC decline was not statistically significant. Overall, the drug was well-tolerated, and only minor side effects were reported. Nevertheless, there was a high level of dissatisfaction with the drug, 70.6% at 48 weeks, and 88.9% at 72 weeks. The more significant decline in the ALSFRS-R values over the clinical trials could be explained by the concomitant use of riluzole in this study, with 88.2% [22]. 

Israel 

In the Israeli study, 22 patients were treated with edaravone, and 71 patients untreated with edaravone without using the specific criteria that were used in the initial clinical trials were proved to be effective [8,13]. Most of the patients were on a stable dose of riluzole and received the drug for at least six months. Two groups were compared in the study; the ALS patients treated with edaravone and the control group of all edaravone-untreated ALS patients. We compared the two groups' rates of decline of ALSFRS-R, manual muscle testing (MMT), and survival as the time to death or tracheostomy. No difference was found in the comparisons of any of the parameters. Of course, it is essential to point out that none of the patients fulfill the criteria of phase III clinical trials where edaravone seems to be more productive [8]. The authors did not find that surprising because it is expected that the phase III clinical trial's criteria are only fulfilled by 7% of patients with ALS [23]. Treated patients had higher death rates, but this was not statistically significant. Another interesting observation was that one-third of patients presented respiratory complications within hours following infusions, and these patients initially had lower FVC scores at baseline [23].

Italy

Two studies after the approval of edaravone were conducted in Northern Italy [24]. Both studies used the same selection criteria as the MCI-186-J19 study such as being older than 18 years, a score of two or more in all of the items of ALSFR-R, disease duration no longer than two years, decrease in the ALSFRS-R score of 1-4 during a 12-week observation period between screening and baseline, diagnosis of clinically "probable" or "definitive" ALS according to revised El Escorial criteria, and FVC 80% [8]. The first study was conducted among 31 patients, the objective of which was to determine changes in ALSFR-R score, FVC value, and the Medical Research Council score between patients treated and not treated with edaravone. Nevertheless, there were no functional differences in any of the parameters [24].

The second study was conducted among a larger cohort of 231 subjects. Among the limitations of the study was not having a parallel control group. That is why it was decided to use a database of patients with the same inclusion/exclusion criteria as the one used in the study. The study confirmed the results of the previous Italian study [24]. That is, the second Italian study did not show significant differences in disease progression and respiratory function. Overall, edaravone was well-tolerated [25]. To assess survival, they studied whether edaravone delays reaching FVC of 60% or if an ALSFRS-R defines as D-50, which is half of the functional scale. Nevertheless, no differences were found in the decline of these parameters in both groups [25].

Comparing the Clinical Trial With the Post-Marketing Experience 

We describe the rate of decline of the ALSFR-R of patients in the clinical trials. We begin by comparing the open-label studies in Korea and Israel with the clinical trial MCI-186 because they have almost the same inclusion/exclusion criteria. An important detail is the lack of a control group in the Korean study. In Table 3, we compare the FVC and the decline of the ALSFR-R score for six months in the Korean and Israeli studies with study MCI-186 [7,21,23]. 

**Table 3:** Comparison of the decline in ALSFR-S and FVC score between MCI-186 clinical trial and the post-marketing experience in Israel and Korea. ALSFRS-R: Revised amyotrophic lateral sclerosis functional rating scale. LS mean: Least squares mean. This is a mean estimated from a linear model. SE: Standard error. FVC: Forced vital capacity. ΔFS: Rate of disease progression for ALS as a significant predictor of survival in ALS. MCI-186: Is the name of the study from Japan with two extensions namely MCI-186-17 and MCI-186-19, respectively.

Author, year [reference]	Study (country)	ALSFRS-R score progression in placebo group [time]	ALSFRS-R score progression in treatment group [time]	p (value)	FVC values progression in placebo group [time]	FVC values progression in treatment group [time]	p (value)
Abe et al., 2014 [7]	MCI-186 clinical trial (Japan)	LS mean ± SE: 6.35 ± 0.84 [24 weeks]	LS mean ± SE: 5.70 ± 0.85 [24 weeks]	0.411	LS mean ± SE: 17.49 ± 2.39 [24 weeks]	LS mean ± SE: 14.57 ± 2.41 [24 weeks]	0.1993
Abraham et al., 2018 [23]	Israeli study	ΔFS disease progression rate ALSFRS-R: 0.87± 1 [24 weeks]	ΔFS disease progression rate ALSFRS-R: 0.92 ± 0.84 [24 weeks]	0.83	ΔFS disease progression rate FVC: 2.04 ± 3.66% [24 weeks]	ΔFS disease progression rate FVC: 2.06 ± 2.15% [24 weeks]	0.99
Park et al., 2020 [21]	Korea study	Data not provided, no control	ΔFS disease progression rate ALSFRS-R: 5.75 ± 6.07 [24 weeks)	Data not provided but not statistically significant	Data not provided, no control	ΔFS disease progression rate FVC: 8.7 ± 17.0% [24 weeks]	Data not provided but not statistically significant

The two studies conducted in Kuwait and Italy used almost the same inclusion/exclusion criteria as the study MCI-186-J19, where edaravone proved to be effective [8]. In both studies, a database of patients was used in order to have a control group [22,25]. The first study in Italy lasted 24 weeks, while the second lasted 48 weeks, the same as study MCI-186-17. The study MCI-186-17 was an extension of the study MCI-186 [25]. We only used the second study in Italy because it has a bigger patient pool than in the first study. We compared the second study of Italy and the Kuwait study to the group that received edaravone for the first six months and used the open-label portion of the clinical trial (E-E group), the MCI-186-17 trial, with study MCI-186-J19 [22,25]. It is important to note that the MCI-186-J19 study was the first to show efficiency in early-ALS patients [8]. The study in Kuwait lasted 72 weeks, so we do not have a clinical trial to compare to that portion of the study [22]. Kuwait's study duration is significant because it is the only publication available to see edaravone's extended effect in patients with ALS. Tables 4, 5 summarize the comparison between these studies and clinical trials [8,13,22,25].

**Table 4:** Comparison of the decline in ALSFRS-R in clinical trials (MCI-186-19 and MCI-186-17) of edaravone and the post-marketing experience in Italy and Kuwait. ALSFRS-R: Revised amyotrophic lateral sclerosis functional rating scale. LS mean: Least squares mean. This is a mean estimated from a linear model. SE: Standard error. FVC: Forced vital capacity. ΔFS: Rate of disease progression for ALS as a significant predictor of survival in ALS. E-E group: Group of patients who were taking edaravone for 24 weeks, and we are giving the same drug for an additional 24 weeks. P-E group: Group of patients receiving placebo in the first phase of the study and then switched to the edaravone. MCI-186: Is the name of the study from Japan with two extensions namely MCI-186-17 and MCI-186-19, respectively.

Author, year [reference]	Study (country)	Progression of ALSFRS-R score in the treatment group [time]			Progression of ALSFRS-R score in the control group [time]		p (value) [24 weeks]	p (value) [48 weeks]	p (value) [72 weeks]
Takei et al., 2017 [8]	MCI-186-J19 clinical trial (Japan)	Adjusted mean LS mean ± SE: 5.01 ± 0.64 (treatment) [24 weeks]			Adjusted mean LS mean ± SE: 7.50 ± 0.66 (control) [24 weeks]		0.0013	No data	No data
Takahashi et al., 2017 [13]	MCI-186-17 clinical trial (Japan)	Adjusted mean LS mean ± SE: 4.58 ± 1.55 (E group) [24 weeks]	Adjusted mean LS mean ± SE: 4.22 ± 1.04 (E-E group) [48 weeks]	Adjusted mean LS mean ± SE: 7.02 ± 1.39 (P-E group) [48 weeks]	-5.01 ± 0.64 (Placebo) [24 weeks]		p = 0.0270	p = 0.0719	No data
Lunetta et al., 2020 [25]	Italian study #2	ΔFS disease progression rate ALSFRS-R: 5.0 [2.0–9.0] [24 weeks]	ΔFS disease progression rate ALSFRS-R: 9.0 [5.0–15.0] [48 weeks]		ΔFS disease progression rate ALSFRS-R: 3.0 [1.0–7.0] [24 weeks]	ΔFS disease progression rate ALSFRS-R: 8.0 [3.0–14.0] [48 weeks]	p = 0.01	p = 0.56	No data
Ismail et al., 2020 [22]	Kuwait study	ΔFS disease progression rate ALSFRS-R: 1.6 ± 1.9 [24 weeks]	ΔFS disease progression rate ALSFRS-R: 3.8 ± 3.0 [48 weeks]	ΔFS disease progression rate ALSFRS-R 7.9 ± 6.6 [72 weeks]	(No control)		p = 0.523	p = 0.055	p = <0.001

**Table 5:** Comparison of the decline in ALSFRS-R and FVC values in MCI clinical trials (MCI-186-19 and MCI-186-17) of edaravone and the post-marketing experience in Italy and Kuwait (Part 2). ALSFRS-R: Revised amyotrophic lateral sclerosis functional rating scale. LS mean: Least squares mean. This is a mean estimated from a linear model. SE: Standard error. FVC: Forced vital capacity. ΔFS: Rate of disease progression for ALS as a significant predictor of survival in ALS. E-E group: Group of patients who were taking edaravone for 24 weeks, and we are giving the same drug for an additional 24 weeks. P-E group: Group of patients receiving placebo in the first phase of the study and then switched to the edaravone. MCI-186: Is the name of the study from Japan with two extensions namely MCI-186-17 and MCI-186-19, respectively.

Author, year [reference]	Study (country)	Progression of FVC values in treatment group [time]			Progression of FVC values in control group [time]		p (value) [24 weeks]	p (value) [48 weeks]	p (value) [72 weeks]
Takei et al., 2017 [8]	MCI-186-J19 clinical trial (Japan)	NO EVALUATION							
Takahashi et al., 2017 [13]	MCI-186-17 clinical trial (Japan)	Adjusted mean LS mean ± SE: 5.93 ± 0.82 [24 weeks]	Adjusted mean LS mean ± SE: 4.11 ± 0.79 (E-E group) [48 weeks]	Adjusted mean LS mean ± SE: 5.13 ± 0.68 (P-E group) [48 weeks]	Adjusted mean LS mean ± SE: 4.82 ± 0.86 (Placebo) [24 weeks]		p = 0.1548 [24 weeks]	p = 0.2884 [48 weeks]	No data
Lunetta et al., 2020 [25]	Italian study #2	ΔFS disease progression rate FVC: 14.5 [3.0-33.0] [24 weeks]	ΔFS disease progression rate FVC: 20.0 [11.5-31.0] [48 weeks]		ΔFS disease progression rate FVC: 8.2 [1.0-18.0] [24 weeks]	ΔFS disease progression rate FVC: 22.3 [8.6-40.1] [48 weeks]	p = 0.01 [24 weeks]	p = 0.85 [48 weeks]	No data
Ismail et al., 2020 [22]	Kuwait study	ΔFS disease progression rate FVC: 7.44 ± 3.6 [24 weeks]	ΔFS disease progression rate FVC: 13.03 ± 3.9 [48 weeks]	ΔFS disease progression rate FVC: 17.24 ± 4.1 [72 weeks]	(No control)		p = 0.342 [24 weeks]	p = 0.236 [48 weeks]	p = <0.089 [72 weeks]

The clinical trials received some criticism. First, the selection of patients was very restricted. With the criteria applied, only a small number of patients would be eligible to use the drug. Second, the studies' durations were too short compared to the average in which European studies test their drugs (12-18 months). Third, the decreasing rate of ALSFR-R scores is not a measure of survival, so long-term survival cannot be proven [5]. It is imperative to note that most clinical trial patients were also using riluzole at the time of diagnosis. This was also the case in the post-marketing experience. Further research needs to be carried out in order to see the possible additive effect of riluzole on edaravone to treat ALS.

According to the Northeast ALS Consortium database, the ALSFR-R score decreases at -0.92 units per month with a small variance [26]. With this parameter in consideration, the Italian and Israeli studies were unsuccessful, and the studies in Kuwait and Korea showed a moderate benefit with the use of edaravone. The limitation of the study was minimal in the post-marketing experience. Physicians are also prescribing the medication without using the inclusion/exclusion criteria of the MCI-186-J19 study [8], where clinical efficiency was proven. 

## Conclusions

Edaravone has been demonstrated as an effective drug for patients with ALS at an early stage in clinical trials. Other studies have shown moderate benefits for these patients or failed to establish a good clinical outcome. The post-marketing experience in Kuwait and Korea had moderately beneficial results in decreasing the progression of the disease. On the other hand, studies in Italy and Israel failed to show improvement or decrease progression of ALS with edaravone. There is limited information about the efficacy of edaravone in the United States and Argentina due to the limited number of reports about the efficacy of edaravone. This is why more post-marketing experience with edaravone and its impact in the clinical setting need to be reported to get a better perspective of edaravone's effectiveness. 
